# Research on Adaptive Identification Technology for Rolling Bearing Performance Degradation Based on Vibration–Temperature Fusion

**DOI:** 10.3390/s25154707

**Published:** 2025-07-30

**Authors:** Zhenghui Li, Lixia Ying, Liwei Zhan, Shi Zhuo, Hui Li, Xiaofeng Bai

**Affiliations:** 1 College of Mechanical and Electrical Engineering, Harbin Engineering Univesity, Harbin 150500, China; lizhenghui1990@126.com; 2 Aero Engine Corporation of China Harbin Bearing Company, Ltd., Harbin 150500, China; zhuoshi8125@163.com (S.Z.); aecchzlihui@163.com (H.L.); 3 School of Instrument Science and Engineering, Harbin Institute of Technology, Harbin 150001, China; 22b901042@stu.hit.edu.cn

**Keywords:** rolling bearing, feature set, vibration, temperature, life evolution

## Abstract

To address the issue of low accuracy in identifying the transition states of rolling bearing performance degradation when relying solely on vibration signals, this study proposed a vibration–temperature fusion-based adaptive method for bearing performance degradation assessments. First, a multidimensional time–frequency feature set was constructed by integrating vibration acceleration and temperature signals. Second, a novel composite sensitivity index (CSI) was introduced, incorporating the trend persistence, monotonicity, and signal complexity to perform preliminary feature screening. Mutual information clustering and regularized entropy weight optimization were then combined to reselect highly sensitive parameters from the initially screened features. Subsequently, an adaptive feature fusion method based on auto-associative kernel regression (AFF-AAKR) was introduced to compress the data in the spatial dimension while enhancing the degradation trend characterization capability of the health indicator (HI) through a temporal residual analysis. Furthermore, the entropy weight method was employed to quantify the information entropy differences between the vibration and temperature signals, enabling dynamic weight allocation to construct a comprehensive HI. Finally, a dual-criteria adaptive bottom-up merging algorithm (DC-ABUM) was proposed, which achieves bearing life-stage identification through error threshold constraints and the adaptive optimization of segmentation quantities. The experimental results demonstrated that the proposed method outperformed traditional vibration-based life-stage identification approaches.

## 1. Introduction

Rolling bearings, as critical components of rotating machinery, directly influence the operational reliability and service life of equipment [1]. Traditional bearing life assessment methods primarily rely on a vibration signal analysis, which monitors degradation through time-domain and frequency-domain feature extraction combined with fault frequency identification [2,3]. However, single-channel vibration signals are susceptible to noise interference under complex operating conditions and exhibit limited characterization capabilities for early-stage weak faults and multi-mode composite damage [4,5], leading to insufficient model generalizability. In recent years, multi-source signal fusion techniques have significantly enhanced degradation feature representation by integrating multi-physics data such as the vibration, temperature, and slippage [6,7]. For instance, the research reported in [8] demonstrates that temperature signals effectively reflect the gradual transition process of bearing friction and lubrication states, complementing the transient impact characteristics of vibration signals. Reference [9] combined acoustic and vibration data to achieve an over 99% diagnostic accuracy across all signal-to-noise ratio (SNR) conditions, exhibiting an exceptional robustness and noise immunity in both noisy and high-SNR environments. Nevertheless, the high dimensionality and redundancy of multi-source features escalate the modeling complexity, urgently demanding efficient feature fusion and screening mechanisms [10]. Addressing the inherent non-stationarity of vibration signals under evolving defect conditions remains particularly challenging. Recent approaches, such as the dynamic modeling framework proposed by Galli et al. for non-stationary bearing vibration signals [11], offer promising avenues for simulating complex degradation behaviors.

In the field of feature extraction and fusion, time–frequency analysis methods such as wavelet packet decomposition (WPD) and empirical mode decomposition (EMD) have been widely applied to capture the local characteristics of nonlinear signals [12,13]. For instance, reference [14] utilized wavelet packet energy entropy to construct multidimensional feature sets but failed to address the interference from inter-feature correlations; reference [15] proposed an adaptive feature fusion (AFF) strategy to compress the data dimensionality by dynamically adjusting the feature weights, although its long-term dependency on degradation trends remained insufficient. The auto-associative kernel regression (AAKR) method quantifies residuals through health space mapping, demonstrating a high sensitivity in rolling bearing life predictions [16], yet its computational complexity has hindered real-time applicability [17]. To address these limitations, this study adopted a dual-stage AFF–AAKR fusion framework, integrating spatial compression with a temporal residual analysis to balance the computational efficiency and the degradation characterization capability.

Feature sensitivity screening constitutes a critical step in optimizing the model performance. The existing studies have predominantly employed single-indicator metrics (e.g., monotonicity, trendability) to evaluate feature-degradation correlations [18], yet they have overlooked the synergistic effects of multidimensional indicators. For instance, reference [19] introduced a mutual information-based feature-clustering method but failed to incorporate regularization constraints to suppress redundancy; reference [20] jointly utilized trendability and complexity metrics to screen sensitive features, yet its linear weighting strategy struggled to balance nonlinear relationships among the indicators. To address these limitations, this study designed a composite sensitivity index (CSI) that integrates the trend persistence index (TPI), the trend monotonicity index (TMI), and the signal complexity index (SCI). By implementing a product constraint mechanism, the CSI achieves multi-indicator synergy optimization. Furthermore, the framework combines K-means++ clustering with an L1-regularized entropy weight allocation to effectively extract low-redundancy high-sensitivity parameter sets from high-dimensional features.

Health state partitioning models must adapt to the nonlinear and phased characteristics of bearing degradation. Traditional methods, such as hidden Markov models (HMMs) and Gaussian mixture models (GMMs), rely on prior distribution assumptions and are prone to mis-segmentation under complex degradation patterns [21,22,23,24,25,26]. To overcome these limitations, this study developed a dual-criteria adaptive bottom-up merging (DC-ABUM) algorithm, which integrates fitting error thresholds and segment quantity constraints to achieve the adaptive three-stage partitioning of degradation curves. This approach significantly enhances the turning point identification accuracy by dynamically optimizing the segmentation granularity based on a residual analysis and statistical significance testing.

Furthermore, existing health indicator (HI) evaluation systems predominantly focus on singular performance metrics (such as monotonicity or correlation), lacking a comprehensive quantitative framework [27,28]. Although reference [29] proposed a robustness indicator based on trend-residual decomposition, its weight assignment relied on empirical settings. Reference [30] employed detrended fluctuation analysis (DFA) to assess the long-term correlation of the HI but did not integrate it with short-term dynamic characteristics. This paper constructs a comprehensive index (CI) encompassing monotonicity, correlation, and robustness. Through weighted fusion, the CI quantifies the overall performance of the HI, providing a unified benchmark for method comparison.

To address the aforementioned challenges, this paper proposes a multi-source signal fusion and adaptive optimization framework for rolling bearing health monitoring. The framework employs a composite sensitivity index (CSI) with a two-tier screening mechanism (combining mutual information clustering and L1-regularized entropy weighting) to eliminate redundant features while preserving degradation-sensitive parameters. A dynamic feature fusion model (AFF–AAKR) is further designed to integrate spatial compression (via adaptive feature fusion) and temporal residual analysis (via auto-associative kernel regression), constructing a high-performance health indicator (HI) with minimized redundancy and maximized degradation information. Additionally, the entropy weight method dynamically allocates weights to vibration and temperature signals based on their information entropy contributions, ensuring optimal multi-source fusion. Finally, the DC-ABUM-based health state partitioning model is developed to precisely delineate the degradation stages (e.g., normal, incipient fault, severe degradation) by jointly minimizing fitting errors and controlling stage transitions, thereby reflecting the actual degradation process with higher fidelity.

The remainder of this paper is organized as follows: Section 2 introduces foundational theories and related methodologies. Section 3 details the proposed two-tier feature selection methodology (CSI-based screening and entropy-weighted fusion) and the DC-ABUM-driven health state partitioning model. Section 4 validates the proposed framework through accelerated bearing degradation experiments and benchmarks its performance against state-of-the-art methods in terms of HI smoothness, stage identification accuracy, and computational efficiency. Finally, Section 5 concludes the paper and suggests future research directions.

## 2. Theoretical Methodology

### 2.1. Feature Extraction Based on Vibration Acceleration Signals

When surface damage occurs in bearing components (e.g., inner/outer races or rolling elements), transient shock pulses are generated at mechanical contact interfaces. These micro-impact events induce characteristic harmonic components in the vibration signal spectrum, whose frequency parameters exhibit a strict mathematical correspondence with specific fault types. By constructing a fault-sensitive feature set, this mechanism enables full-lifecycle monitoring of bearing operational states and precise fault mode identification. In this study, feature sets are systematically developed across three domains: time-domain, frequency-domain, and time–frequency-domain analyses.

(1) Time-Domain Features

Time-domain features reveal the operational states and fault patterns of bearings by directly analyzing the statistical characteristics of vibration signals along the time axis. These features are categorized into two groups: dimensional statistical parameters, such as the mean, variance, and root mean square (RMS), which evaluate the overall amplitude level and energy distribution of the signal, and dimensionless statistical parameters, including kurtosis, crest factor, and waveform factor. The latter demonstrate superior noise immunity due to their insensitivity to amplitude scaling and baseline shifts, offering significant advantages in detecting early-stage weak faults in bearings. The time-domain features and their definitions are systematically cataloged in Table 1.

(2) Frequency-domain features

Frequency-domain features are derived by mapping vibration signals into the frequency dimension via fast Fourier transform (FFT), enabling the precise localization of fault-related characteristic frequencies and their energy distribution patterns. Core parameters, such as the mean frequency and frequency-domain root mean square (FRMS), are utilized to identify fault types and their corresponding spectral signatures. The frequency-domain feature extraction process and parameters are detailed in Table 2.

(3) Time–Frequency Feature

Wavelet packet analysis (WPA) enables the multiscale characterization of signal time–frequency properties. The relationships between the scaling function ϕ(t) and wavelet function ψ(t) are expressed as

(1) $$\left\{\begin{array}{l} \phi (t)=\sqrt{2}\sum_{n}h_{n}\phi (2t-n)\\ \psi (t)=\sqrt{2}\sum_{n}g_{n}\phi (2t-n) \end{array}\right.,$$

where $h_{n}$ and $g_{n}$ denote the coefficients of the low-pass and high-pass filters, respectively, t represents the time variable, and n is the decomposition level index.

The wavelet packet function family $\left\{\omega_{i,j,k}\left(t\right)\right\}$ is generated through recursive decomposition of the scaling space $V_{0}$ and wavelet space $W_{0}$:

(2) $$\left\{\begin{array}{l} V_{i+1}=V_{i}⊕W_{i}\\ W_{i+1}=W_{i}⊕W_{i}^{\prime } \end{array}\right..$$

Extending this to wavelet packets yields

(3) $$\left\{\begin{array}{l} \omega_{2n}(t)=\sum_{k}h_{k}\omega_{n}\phi (2t-k)\\ \omega_{2n+1}(t)=\sum_{k}g_{k}\omega_{n}\phi (2t-k) \end{array}\right..$$

Here, k is an integer index for the wavelet packet nodes. The decomposition and reconstruction formulas are defined as

(4) $$\left\{\begin{array}{l} d_{i+1}^{2n}=\sum_{k}h_{k-2m}d_{l}^{n}\\ d_{i+1}^{2n+1}=\sum_{k}g_{k-2m}d_{l}^{n} \end{array}\right.$$

(5) $$d_{i}^{n}=\sum_{m}{\tilde{h}}_{m-2k}d_{i+1}^{2n}+\sum_{m}{\tilde{g}}_{m-2k}d_{i+1}^{2n+1},$$

where $d_{i}^{n}$ represents the wavelet packet coefficients at the *i*-th decomposition level and *n*-th node, with $\tilde{h}$ and $\tilde{g}$ being the reconstruction filters.

Based on Parseval’s theorem, the signal energy can be represented as the sum of squared wavelet packet coefficients:

(6) $${\int \left|x\left(t\right)\right|}^{2}dt={\sum_{i,j,k}\left|d_{i,j,k}\right|}^{2}.$$

After *i*-level WPA decomposition, the signal is divided into $2^{i}$ frequency bands. The energy of each band is calculated using the RMS formulation established in Equation (7):

(7) $$E_{n}=\frac{1}{N_{n}}{\sum_{k=1}^{N_{n}}\left|d_{i,n}\left(k\right)\right|}^{2}.$$

**Figure 1:**
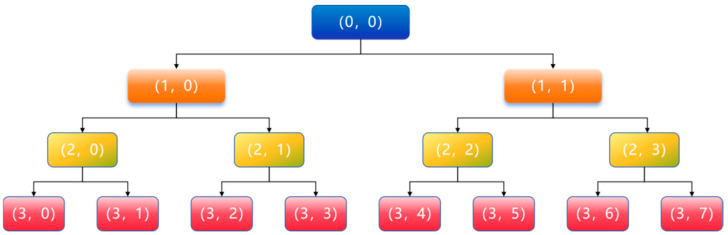
The wavelet packet decomposition process when *i* = 3.

Figure 1 illustrates the decomposition process for *i* = 3, where the original signal is progressively split into eight frequency bands.

### 2.2. Feature-Based AFF–AAKR Fusion

The adaptive feature fusion (AFF) method is a feature fusion approach that constructs a health indicator (HI) through weighted averaging. Its core principle involves dynamically adjusting weights based on the correlations among features to reflect their degradation trends over time [14]. Specifically, assuming a bearing has n features, the feature set is denoted as F, and the feature vector length is m. The observation matrix *X*, composed of n features, has a dimension of m×n. For the feature value $x_{t}$ at time t, its average distance $d_{t}$ to other features at time t is calculated as

(8) $$d_{t}=\frac{1}{n-1}\sum_{i=1,i\neq t}^{n}\left\|x_{t}-x_{i}\right\|.$$

A smaller $d_{t}$ indicates stronger correlations between $x_{t}$ and other features, resulting in a larger weight $w_{t}$:

(9) $$w_{t}=\frac{1}{d_{t}+\varepsilon },$$

where ε is a smoothing parameter. The HI curve generated by the AFF algorithm is

(10) $$HI_{t}=\sum_{i=1}^{n}w_{i}x_{i}.$$

The AFF method offers computational efficiency and spatial compression but often inadequately reflects degradation trends. To enhance degradation characterization, auto-associative kernel regression (AAKR) is adopted over alternative regression methods for three key reasons:

(1) Nonlinear Mapping: Unlike linear regression (e.g., PCA), AAKR captures nonlinear relationships between observation vectors and health baselines through kernel functions, which is critical for complex degradation processes.
(2) Residual Sensitivity: Compared to Gaussian process regression (GPR), AAKR directly quantifies deviations via Euclidean residuals, providing clearer degradation quantifiers than probabilistic outputs.
(3) Computational Feasibility: While long short-term memory (LSTM) networks model temporal dependencies effectively, they require large labeled datasets and extensive training. AAKR avoids these constraints with minimal training overhead.

Furthermore, the bearing test data in this study exhibit complex non-stationary degradation patterns requiring nonlinear modeling, benefit from direct residual-based degradation quantifiers, and are derived from limited experimental runs, making AAKR’s nonlinear capability, residual sensitivity, and computational efficiency particularly well-suited.

AAKR maps observation vectors to a health space and constructs HI curves by calculating residuals between observation vectors and reconstructed signals [15]. For a rolling bearing with p monitoring parameters, the observation vector $y_{t}$ at time t is expressed as

(11) $$y_{t}=\left[y_{t1},y_{t2},\cdot \cdot \cdot ,y_{tp}\right].$$

The health space matrix H, composed of m health vectors, is defined as

(12) $$H=\left[h_{1},h_{2},\cdot \cdot \cdot ,h_{m}\right].$$

The mapping of ${\hat{y}}_{t}$ in the health space is

(13) $${\hat{y}}_{t}=\sum_{i=1}^{m}w_{i}h_{i},$$

where the weights $w_{i}$ are calculated as

(14) $$w_{i}=\frac{K(\left\|y_{t}-h_{i}\right\|)}{\sum_{j=1}^{m}K(\left\|y_{t}-h_{j}\right\|)}.$$

The kernel function K typically employs a Gaussian form:

(15) $$K\left(d\right)=\exp\left(-\frac{d^{2}}{2\sigma^{2}}\right).$$

The HI value at time t is the Euclidean distance between $y_{t}$ and its reconstructed vector ${\hat{y}}_{t}$:

(16) $$HI_{t}=\left\|y_{t}-{\hat{y}}_{t}\right\|.$$

The AAKR method demonstrates superior performance in constructing health indicator (HI) curves that better align with bearing degradation trends, while quantitatively measuring the temporal discrepancies between observation matrices and health matrices. However, this method exhibits significant computational overhead, particularly when processing large initial datasets, due to the extensive vector operations involved.

To integrate the strengths of both AFF and AAKR methods while mitigating their limitations, this study proposes an AFF–AAKR feature fusion approach. The method first employs AFF-based spatial compression to preliminarily reduce the multi-dimensional feature matrix X into a one-dimensional feature vector Z, thereby effectively decreasing the data volume.

(17) $$Z=\sum_{i=1}^{n}w_{i}x_{i}$$

Subsequently, AAKR further fuses the compressed vector Z in the temporal domain by quantifying residuals between observation vectors and health space reconstructions.

(18) $$HI_{t}=\left\|Z_{t}-{\hat{Z}}_{t}\right\|,$$

where ${\hat{Z}}_{t}$ is the reconstructed value of $Z_{t}$ in the health space. This dual fusion strategy not only resolves AAKR’s computational inefficiency but also enhances HI curve performance.

### 2.3. Comprehensive Health State Evaluation of Bearings via Multi-Information Fusion Using Entropy Weight Method

The entropy weight method (EWM) is a multi-criteria weighting approach that integrates subjective and objective factors. It dynamically adjusts weights by quantifying indicator variability and is widely applied in engineering decision-making and condition assessment [31]. The core workflow comprises four key steps: data standardization, information entropy calculation, weight determination, and comprehensive evaluation.

Significant differences in data dimensions and ranges necessitate standardization to eliminate scale effects and enhance comparability. Common methods include the following:

Min–max normalization, which linearly maps data to the [0, 1] interval:

(19) $$x_{\mathrm{ij}}^{\prime }=\frac{x_{ij}-\min(x_{j})}{\max(x_{j})-\min(x_{j})}.$$

This method is straightforward but sensitive to outliers, potentially distorting data distributions.

Z-score standardization, which transforms data using the mean and standard deviation:

(20) $$x_{\mathrm{ij}}^{\prime }=\frac{x_{ij}-\mu_{j}}{\sigma_{j}},$$

where $\mu_{j}$ is the mean, and $\sigma_{j}$ is the standard deviation. This approach preserves outlier information and offers superior stability for complex data distributions.

Information entropy reflects the disorder degree of indicator data: lower entropy indicates greater variability and higher information content. The steps are as follows:

Probability proportion: For standardized data, compute the proportion of the *i*-th sample under the *j*-th indicator:

(21) $$p_{ij}=\frac{x_{ij}^{\prime }}{\sum_{i=1}^{n}x_{ij}^{\prime }}.$$

Entropy value: Calculate the entropy $E_{j}$ for the *j*-th indicator:

(22) $$E_{j}=-\frac{1}{\ln n}\sum_{i=1}^{n}p_{ij}\ln p_{ij}.$$

When $p_{ij}$ = 0, define $p_{ij}\ln p_{ij}$ = 0 to avoid computational invalidity.

EWM derives weights inversely from entropy values: lower entropy corresponds to higher weights.

Redundancy Calculation: Define the information redundancy degree $d_{j}=1-E_{j}$.

Weight Allocation: Normalize the redundancy to obtain final weights:

(23) $$w_{j}=\frac{d_{j}}{\sum_{j=1}^{m}d_{j}},$$

where m is the total number of samples. The weight vector satisfies $\sum w_{j}=1$, ensuring rationality of the comprehensive evaluation.

The comprehensive health indicator (HI) for each sample is derived by the weighted summation of normalized data:

(24) $$HI_{i}=\sum_{j=1}^{m}w_{j}\cdot x_{ij}^{\prime }.$$

**Figure 2:**
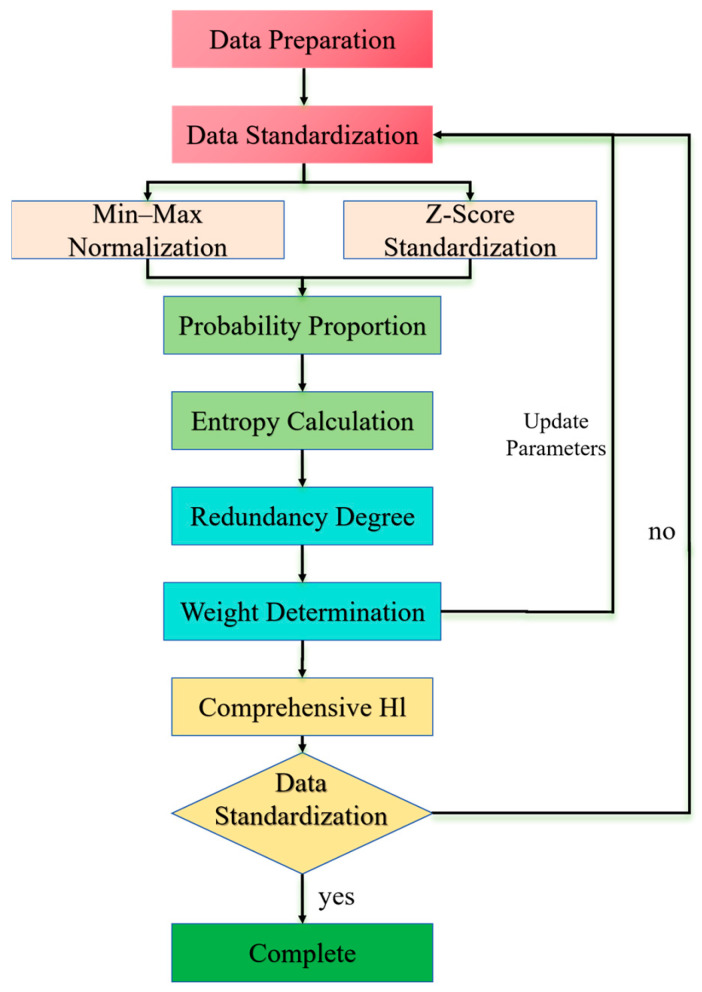
Entropy weight method workflow.

The HI score intuitively reflects the bearing health status: higher values indicate better conditions, providing quantitative support for maintenance decisions. The workflow of the entropy weight method is illustrated in Figure 2.

### 2.4. Comprehensive HI Evaluation Method Based on Monotonicity, Correlation, and Robustness

To systematically evaluate the performance characteristics of health indicators (HI), this study employs a three-dimensional evaluation system based on monotonicity, correlation, and robustness. A comprehensive quantitative metric is formed through weighted fusion.

First, the HI is decomposed into a trend component and a stochastic component:

$$I\left(t\right)=T\left(t\right)+R\left(t\right),$$

where $I\left(t\right)$ represents the HI value at time t, $T\left(t\right)$ denotes the trend component, and $R\left(t\right)$ represents the stochastic component.

Monotonicity measures the consistency of the HI trend evolution, quantified via a sign consistency test of the trend term differences. A higher value indicates a more significant degradation trend, which facilitates the identification of life stages.

(25) $$Mon\left(I\right)=\frac{1}{N-1}\left|\sum_{t}\mu \left(T\left(t+1\right)-T\left(t\right)\right)-\sum_{t}\mu \left(T\left(t\right)-T\left(t+1\right)\right)\right|,$$

where $\mu \left(x\right)=\left\{\begin{array}{c} 1,x>0\\ 0,x\leq 0 \end{array}\right.$, and N is the length of the HI sequence.

Correlation evaluates the association strength between the HI trend component and the physical degradation process of the equipment, measured using a correlation coefficient method to assess the temporal correlation. A higher value reflects superior degradation characterization accuracy.

(26) $$Corr\left(I,t\right)=\frac{\left|N\sum_{t}T\left(t\right)t-\sum_{t}T\left(t\right)\sum_{t}t\right|}{\sqrt{\left[N{\sum_{t}T{\left(t\right)}^{2}-\left(\sum_{t}T\left(t\right)\right)}^{2}\right]\left[N{\sum_{t}t^{2}-\left(\sum_{t}t\right)}^{2}\right]}}$$

Robustness characterizes the HI’s resistance to random disturbances, evaluated through residual error analysis between the trend component and the original HI. A higher value indicates stronger noise resistance and better fluctuation suppression.

(27) $$Rob\left(I\right)=\frac{1}{N}\sum_{t}\exp\left(-\left|\frac{R\left(t\right)}{I\left(t\right)}\right|\right)$$

The CI provides a unified quantitative assessment of health indicator performance by integrating monotonicity, correlation, and robustness through weighted fusion. Based on the study by Wu et al. [31], the coefficient vector is set as $\left(\alpha_{1},\alpha_{2},\alpha_{3}\right)=\left(0.2,0.5,0.3\right)$. The CI formulation is given by Equation (28), where higher CI values (0 ≤ CI ≤ 1) indicate superior degradation characterization capability. This metric serves as the primary benchmark for the comparative method evaluation in Section 4.

(28) $$CI=\alpha_{1}Mon\left(I\right)+\alpha_{2}Corr\left(I,t\right)+\alpha_{3}Rob\left(I\right)$$

## 3. Proposed Methodology

### 3.1. Feature Selection Mechanism Based on Multi-Dimensional Sensitivity Evaluation and Composite Index Optimization

The core of the feature selection mechanism lies in constructing a feature space that characterizes the sensitivity of bearing performance degradation, achieving information concentration by eliminating low-sensitivity features. To establish a quantitative association analysis between feature parameters and degradation trends, a sensitivity evaluation system based on statistical properties is required. This study proposes an enhanced dual-index evaluation framework, constructing a multi-dimensional assessment space using the trend persistence index (TPI) and trend monotonicity index (TMI), and further optimizes the comprehensive quantitative evaluation of feature performance by incorporating the signal complexity index (SCI).

The trend persistence index (TPI) quantifies the consistency of consecutive trend directions in a feature sequence, reflecting the stability of the degradation process. Its mathematical formulation is

(29) $$TPI\left(X\right)=\frac{1}{N-1}\sum_{1}^{N-1}\prod \left(\mathrm{sgn}\left(-x_{i}\right)\cdot \mathrm{sgn}\left(x_{i}-x_{i-1}\right)\right),$$

where $\prod \left(\cdot \right)$ is the indicator function (outputs 1 for consecutive same-sign differences, otherwise 0), and N is the sequence length. The TPI value range is [0, 1], with higher values indicating stronger persistence in degradation trends.

The trend monotonicity index (TMI) quantifies the consistency of evolutionary direction in a feature sequence, defined as

(30) $$TMI\left(X\right)=\frac{1}{N-1}\sum_{1}^{N-1}\mathrm{sgn}\left(x_{i+1}-x_{i}\right),$$

where $\mathrm{sgn}\left(\cdot \right)$ is the sign function, and *N* is the feature sequence length. This index reflects the stability of degradation trends through cumulative directional derivatives, with a value range of [0, 1]. Higher values indicate more significant monotonic interpretability of the degradation process.

The signal complexity index (SCI) evaluates the regularity of feature sequences based on sample entropy (SampEn), defined as

(31) $$SCI\left(X\right)=1-\frac{\mathrm{SampEn}\left(X,m,r\right)}{\log\left(N\right)},$$

where $\mathrm{SampEn}\left(m,r\right)$ is the sample entropy value, m is the embedding dimension, and r is the similarity tolerance. Normalization ensures the SCI value range is [0, 1], with higher values indicating lower signal complexity and stronger correlation with degradation regularity.

These three complementary evaluation metrics are integrated into a composite sensitivity index (CSI) for comprehensive feature performance quantification:

(32) $$CSI\left(X\right)=TPI\left(X\right)\cdot TMI\left(X\right)\cdot SCI\left(X\right).$$

Since the TPI, TMI, and SCI are all normalized (range [0, 1]), the CSI value also converges to [0, 1]. The multiplicative operator in CSI strengthens the synergistic constraints among metrics, ensuring a strict positive correlation between CSI levels and the degradation characterization capability of features.

Based on descending CSI rankings, this study employs statistical significance testing to determine the feature selection threshold. By setting an empirical threshold $\theta_{\mathrm{CSI}}$, features satisfying $CSI\left(X\right)\geq \theta_{\mathrm{CSI}}$ are selected to form a sensitive feature subset. This mechanism effectively retains features with strong degradation characterization capabilities, providing a robust feature foundation for subsequent degradation modeling.

### 3.2. Feature Re-Screening Method Based on Fast Clustering and Regularized Information Entropy

After completing sensitivity-based feature selection, the obtained feature subset eliminates low-sensitivity parameters but still suffers from redundancy due to information-homologous features. The overlapping degradation–informative parameter sets significantly increase the risk of the ‘curse of dimensionality’ in feature fusion and lead to exponential growth in computational complexity. To address this, this study proposes a secondary screening mechanism based on fast clustering and regularized information entropy, achieving efficient feature space optimization through adaptive clustering and sparse constraints.

A symmetric similarity matrix is constructed using mutual information to quantify statistical dependencies between features:

(33) $$S_{ij}=1-\frac{I\left(X_{i};X_{j}\right)}{H\left(X_{i},X_{j}\right)},$$

where $I\left(X_{i};X_{j}\right)$ is the mutual information between features, and $H\left(X_{i},X_{j}\right)$ denotes their joint entropy. This design accurately captures nonlinear feature correlations, avoiding the distributional assumptions inherent in traditional correlation coefficients.

Features are partitioned using an enhanced K-means++ algorithm, where the optimal cluster number K is automatically determined via a silhouette–elbow joint criterion:

The elbow method computes the within-cluster sum of squared errors (SSE) for varying K, selecting K at the ‘elbow’ inflection point.

The silhouette coefficient evaluates intra-cluster compactness and inter-cluster separation simultaneously, choosing K that maximizes the silhouette score.

For each feature cluster Ck, an L1-regularized information entropy weight is assigned:

(34) $$W_{i}=\frac{H\left(X_{i}\right)}{\sum H}-\lambda {\left\|W\right\|}_{1},$$

where $H\left(X_{i}\right)$ is the feature’s information entropy, and λ is a sparsity coefficient. Sparse constraints suppress redundant feature weights, retaining parameters with high information density and low correlation.

### 3.3. Dual-Criteria Adaptive Health State Partitioning Model

To address the nonlinear degradation characteristics of rolling bearing health indicator (HI) curves, this study develops a DC-ABUM algorithm. The core idea is to explicitly decouple degradation stages via piecewise linear approximation (PLA).

Algorithm Framework:

Fitting error threshold: Terminate iterations if the maximum segment fitting error exceeds a preset threshold $\varepsilon_{max}$.

Segment number constraint: Terminate optimization when the predefined segment count M is reached. Mathematically, this solves

(35) $$\min\sum_{i=1}^{M}{\left\|HI-{\hat{HI}}_{i}\right\|}_{2}^{2},$$

where ${\hat{HI}}_{i}$ is the linear fit for the *i*-th segment, and ${\left\|\cdot \right\|}_{2}^{2}$ denotes the Euclidean norm.

For any sub-segment $s_{j}=[t_{a},t_{b}]$, its linear model $\hat{HI}(t)=k_{j}t+b_{j}$ is solved via least squares:

(36) $$\underset{k_{j},b_{j}}{\min}=\sum_{t=t_{a}}^{t_{b}}\left(HI(t)-(k_{j}t+b_{j}\right){)}^{2}.$$

The solved slope $k_{j}$ and intercept $b_{j}$ are

(37) $$k_{j}=\frac{\left(t_{b}-t_{a})\sum t\cdot HI(t)-\sum t\cdot \sum HI(t\right)}{(t_{b}-t_{a})\sum t^{2}-{\left(\sum t\right)}^{2}},\;\mathrm{and}\;b_{j}=\frac{\sum HI(t)-k_{j}\sum t}{t_{b}-t_{a}}.$$

The merge cost between adjacent segments $s_{j}$ and $s_{j+1}$ is defined as the incremental fitting error before and after merging:

(38) $$C(s_{j},s_{j+1})=∥HI_{j\cup j+1}-HI_{j\cup j+1}{∥}_{2}^{2}-(∥HI_{j}-{\hat{HI}}_{j}{∥}_{2}^{2}+∥HI_{j+1}-{\hat{HI}}_{j+1}{∥}_{2}^{2}).$$

Lower merge costs indicate higher consistency in linear trends, prioritizing merges to simplify the model.

Step 1: Initialization

Divide the HI sequence of length N into N atomic segments $S=\left\{s_{1},s_{2},\cdot \cdot \cdot ,s_{N}\right\}$, each containing a single data point.

Step 2: Cost Calculation

Compute merge costs $C\left(s_{j},s_{j+1}\right)$ for all adjacent segment pairs and store them in a priority queue Q.

Step 3: Iterative Optimization

Extract the pair $\left(s_{m},s_{m+1}\right)$ with minimal cost from Q and merge them into ${s^{\prime }}_{m}$.

Update Q: Remove costs associated with the original pairs, compute new costs for ${s^{\prime }}_{m}$ with its neighbors, and reinsert into Q.

Terminate if ∣S∣ ≤ M or $\max\left(\varepsilon_{i}\right)\leq \varepsilon_{\max}$.

Step 4: Output

Return the final segmentation $S=\left\{s_{1},s_{2},\ldots ,s_{M}\right\}$, completing health state partitioning.

The pseudocode is presented in Algorithm 1.
**Algorithm 1** Health State Partitioning1: **Input:** HI_sequence, M, $\varepsilon_{\max}$2: **Output:** S (Final Segmentation)3: Initialize:4:   $\;S=\left\{s_{1},s_{2},\cdot \cdot \cdot ,s_{N}\right\}$$,\;\mathrm{where}\;\mathrm{each}\;s_{i}$, is a data point from HI_sequence.5:    Q = Priority Queue of pairs $\left(C\left(s_{j},s_{j+1}\right),s_{j},s_{j+1}\right)$ for all adjacent pairs.6:    $C\left(s_{j},s_{j+1}\right)$= merge cost function $\left(s_{j},s_{j+1}\right)$7: **Iterative Merging:**8: **while **
$\left|S\right|>M$ and $\;\max\left(\varepsilon_{i}\right)>\varepsilon_{\max}$ do 9.         $\;\left(C_{\min},s_{m},s_{m+1}\right)=Q$ 10:
        ${s^{\prime }}_{m}=merge\left(s_{m},s_{m+1}\right)$ {Merge segments}11:         **Update Queue:**12:         Remove old pairs costs: $C\left(s_{m-1},s_{m}\right),C\left(s_{m},s_{m+1}\right),C\left(s_{m+1},s_{m+2}\right)$13:         Insert new costs: $C\left({s^{\prime }}_{m-1},{s^{\prime }}_{m}\right),C\left({s^{\prime }}_{m},{s^{\prime }}_{m+1}\right)$14: **end while**15: **Termination:**16: **if** 
$\left|S\right|\leq M\;$ or $\;\max\left(\varepsilon_{i}\right)\leq \varepsilon_{\max}$ **then**17:    **Stop**18: **end if**19: Output:$S=\left\{s_{1},s_{2},\cdot \cdot \cdot ,s_{M}\right\}$.

### 3.4. Algorithm Implementation Steps

**Figure 3:**
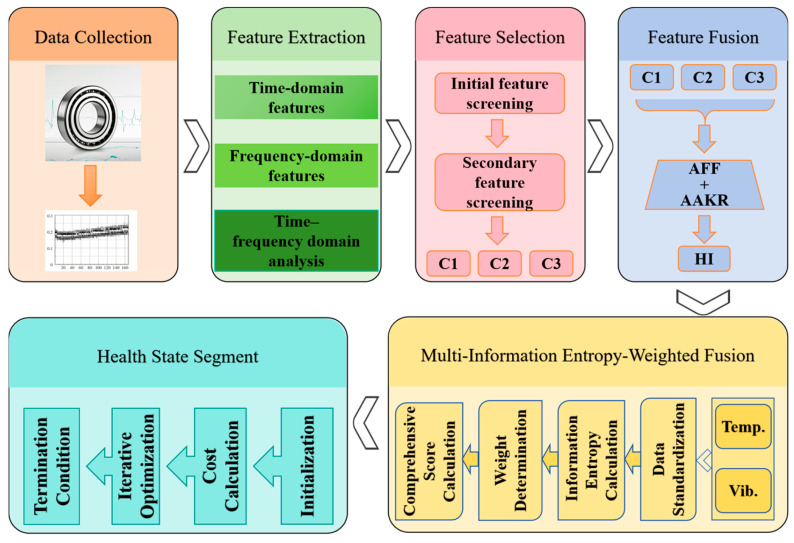
Flow chart of the implementation of proposed algorithm.

The implementation workflow of the proposed algorithm is illustrated in Figure 3. First, the vibration and temperature signals are synchronously acquired with temporal alignment, where the vibration signals are sampled at 25.6 kHz and the temperature signals at 1 Hz. Time-domain statistics, frequency-domain parameters, and wavelet packet energy features (using decomposition level *i* = 3) are extracted from the vibration signals, while temperature signal features including the moving root mean square (RMS) (window size = 60 s) and the gradient magnitude are derived to comprehensively characterize the bearing degradation. Subsequently, a multi-dimensional sensitivity evaluation and composite index optimization-based feature selection mechanism are applied for preliminary feature screening with the CSI threshold τ = 0.3. Then, the fast clustering and regularized information entropy-based re-screening method is employed to achieve efficient optimization and reconstruction of the feature space, where mutual information clustering uses K-means++ (K = 3 clusters), and entropy weighting applies an L1-regularization coefficient λ = 0.1. Next, the AFF method with a smoothing parameter ε = 1 × 10^−6^ dynamically weights and compresses the multi-dimensional features to generate a one-dimensional feature sequence. The AAKR method with a Gaussian kernel bandwidth σ = 0.1 is then used to quantify residuals between observation vectors and the health space, constructing a degradation-sensitive HI curve that balances the computational efficiency and trend characterization. Afterward, information entropy values of vibration and temperature HIs are calculated separately, dynamically weighted, and fused to generate a comprehensive HI curve, enhancing robustness in degradation representation. Finally, the dual-criteria health state partitioning method with a maximum fitting error $\varepsilon_{\max}$ = 0.05 and target segment count M = 3 divides the operational states of the bearing into distinct phases (normal, incipient fault, severe degradation).

## 4. Experiments

### 4.1. Experimental Conditions

**Figure 4:**
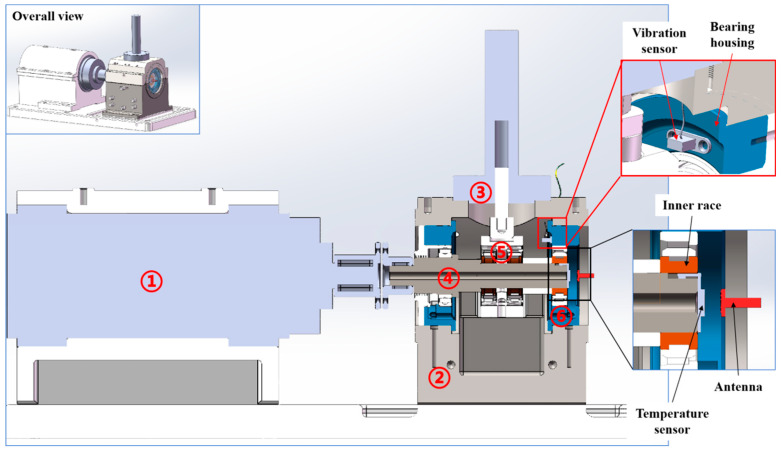
Structure of the test rig and sensor installation positions: ①-motor; ②-test chamber; ③-hydraulic loading cylinder; ④-rotating shaft; ⑤-bearing body; ⑥-bearing body.

The data used in this study were collected from characteristic parameters during accelerated bearing degradation tests. The test bearings were deep groove ball bearings, with a total of 16 sets. The structural parameters are listed in Table 3. Figure 4 illustrates the symmetrically simply-supported bearing tester structure and sensor installation locations. The tester consists of a motorized spindle, test chamber, loading lever, rotating shaft system, load-bearing composite body, test bearings, and bearing housings. Vibration sensors were installed on the end face of the bearing housings, while temperature sensors were pre-embedded in grooves on the shaft and in contact with the inner surface of the inner ring. The sensor specifications are provided in Table 4.

Each bearing set was operated under constant conditions until failure (Table 5).

Signal acquisition used LabVIEW Professional Development System (2023 (64-bit), National Instruments, Austin, TX, USA), while feature extraction, fusion, and modeling were implemented in Python 3.9.

### 4.2. Feature Extraction

**Figure 5:**
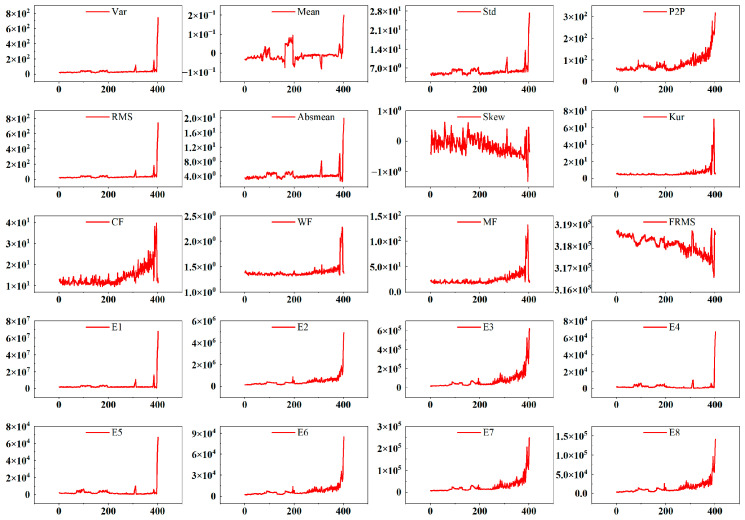
Joint time–frequency characteristic curve.

Before feature extraction, the raw signals underwent basic preprocessing to improve data quality. Vibration signals were smoothed using a low-pass filter with a 10 kHz cutoff frequency to suppress high-frequency noise. Temperature signals were processed with a simple moving average over 30 s intervals to suppress fluctuations and then zero-mean normalized. The vibration (sample rate: 25.6 kHz) and temperature (sample rate: 1 Hz) data were aligned by averaging vibration samples within each 1 s temperature interval. Following this preprocessing, time-domain, frequency-domain, and time–frequency domain features were extracted from the vibration data of each bearing type based on the methodology described in Section 3. Figure 5 shows the extracted feature curves for bearing BG05.

### 4.3. Feature Selection

**Figure 6:**
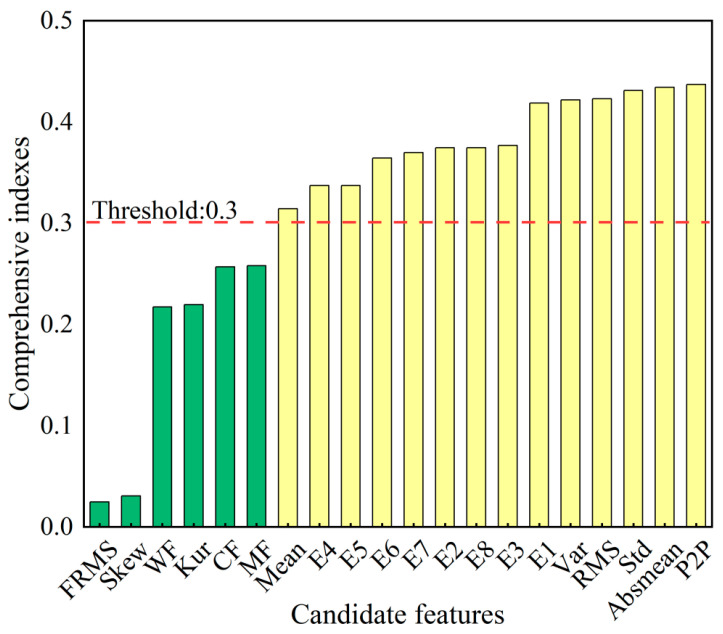
Comprehensive index ranking and selection.

A composite sensitivity index (CSI) was computed for each candidate feature using Equation (32). With an empirical threshold $\theta_{\mathrm{CSI}}$ = 0.3, the normalized CSI rankings and selection results for vibration features are shown in Figure 6. Fourteen features (yellow highlights) were retained:

$$F_{init}=\left\{Mean,E4,E5,E6,E7,E2,E8,E3,E1,Var,RMS,Std,Absmean,P2P\right\}.$$

**Figure 7:**
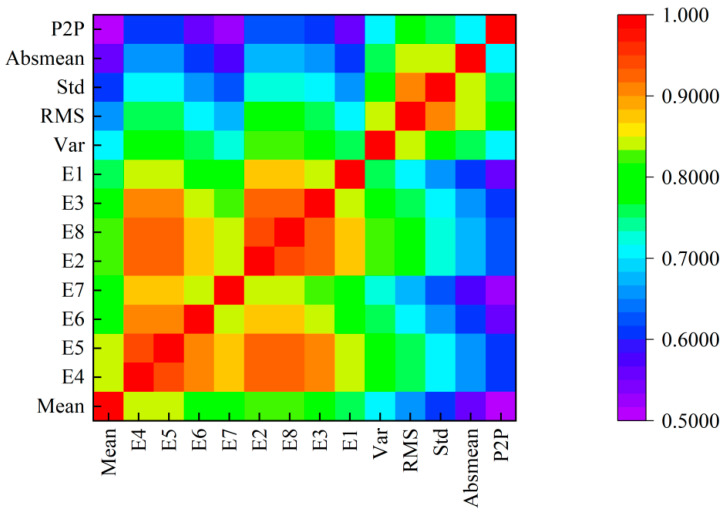
Heatmap of feature similarity matrix.

The feature similarity matrix $S_{ij}$ was calculated via Equation (33) (Figure 7). Lighter hues indicate lower redundancy and darker hues higher redundancy.

**Figure 8:**
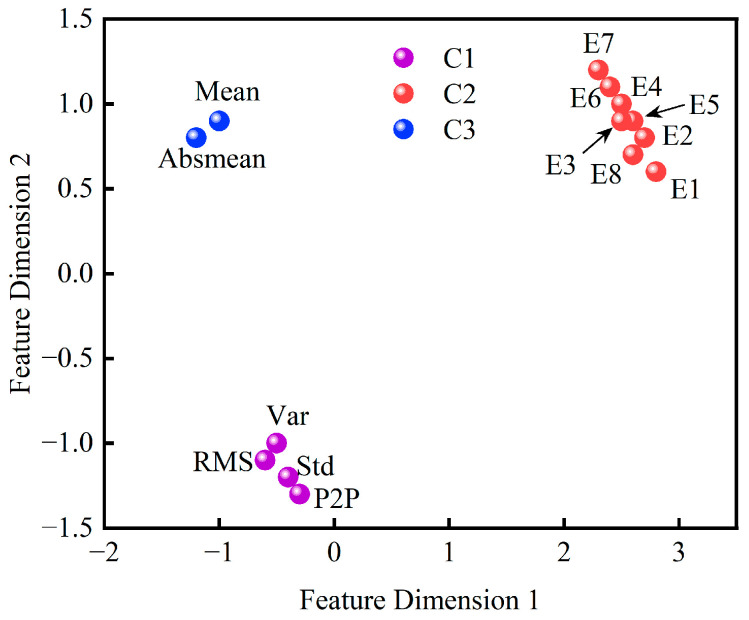
K-means++ scatter plot of clustering results.

K-means++ clustering determined the optimal cluster count K = 3. Figure 8 shows the scatter plot of clustering results based on mutual information similarity, with features partitioned into three categories (Table 6).

Regularized entropy weights per cluster were computed via Equation (34) (Table 7). L1 regularization (λ = 0.1) suppressed the redundant feature weights while emphasizing high-information-density parameters.

The representative features selected per cluster are listed in Table 8.

The final feature subset is defined as

$$F_{\sec}=\left\{Std,E1,Absmean\right\}$$

The screening results for all bearings are summarized in Table 9.

### 4.4. Feature Fusion and Entropy-Based Multi-Information Fusion

Optimized features were fused via the AFF–AAKR method (ϵ = 1 × 10^−6^, Gaussian kernel K = 0.1). The first 10 samples served as the health reference, generating HIs via Equations (8)–(18).

The information entropy of the vibration/temperature HIs was calculated via Equations (19)–(22). The dynamically assigned weights (Equation (23)) are listed in Table 10.

**Figure 9:**
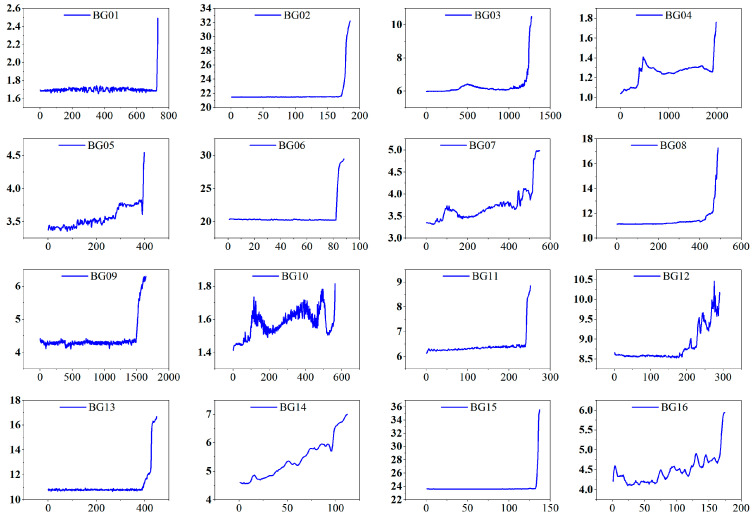
HI curve.

The fused HI curve (Equation (24)) is shown in Figure 9.

### 4.5. Health State Partitioning

**Figure 10:**
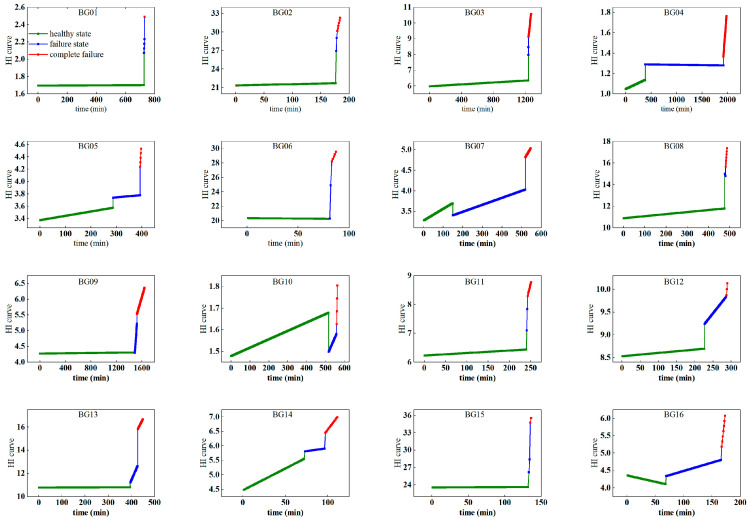
Classification results of health states of 15 experimental bearings.

HIs were partitioned into three states (healthy state/failure state/complete failure) using the DC-ABUM algorithm (M = 3). Health stage divisions for each bearing are shown in Figure 10.

**Figure 11:**
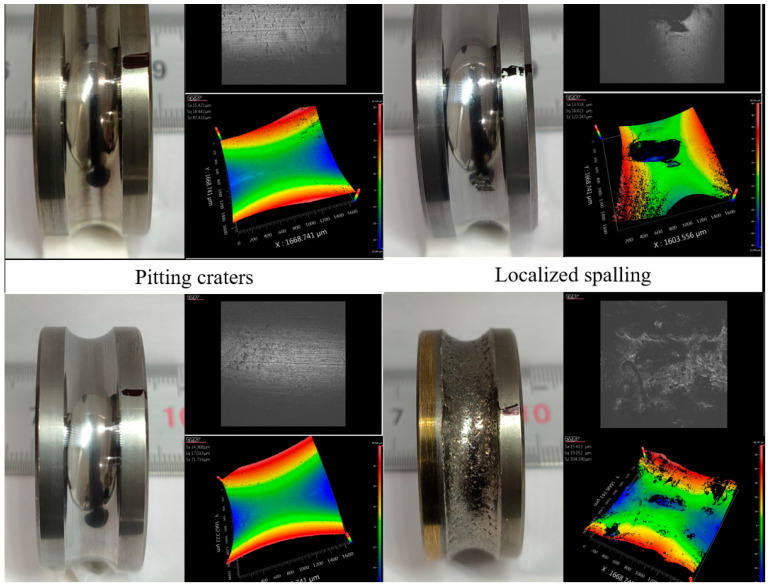
Typical bearing failure modes.

Figure 11 shows typical bearing damage morphologies corresponding to the classified health states.

### 4.6. Results

**Figure 12:**
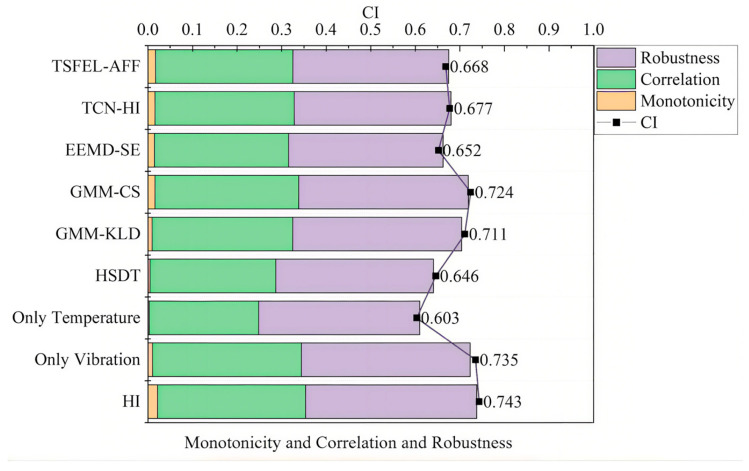
Comparative analysis of health index (HI) construction methods.

To rigorously evaluate the proposed vibration–temperature fused health indicator (HI) methodology, quantitative metrics—including monotonicity, correlation, robustness, and the comprehensive HI index—were employed. The method was benchmarked against nine established approaches: single-source HIs derived from vibration or temperature signals; health state data training (HSDT) [32]; Gaussian mixture model (GMM)-based indicators using Kullback–Leibler divergence (GMM-KLD) [33] and cosine similarity (GMM-CS); ensemble empirical mode decomposition–sample entropy (EEMD-SE) [34]; temporal convolutional network-based HI (TCN-HI) [35]; and time series feature extraction library with adaptive feature fusion (TSFEL-AFF) [36]. Figure 12 compares the performance metrics.

The proposed HI exhibits slightly weaker correlation than the vibration-only HI, yet demonstrates superior performance in other metrics. Specifically, it achieves comprehensive improvements of 1.1%, 23.2%, 15.1%, 4.5%, 2.6%, 13.9%, 9.7%, and 11.2% against the eight comparative methods, demonstrating the effective utilization of degradation information.

**Figure 13:**
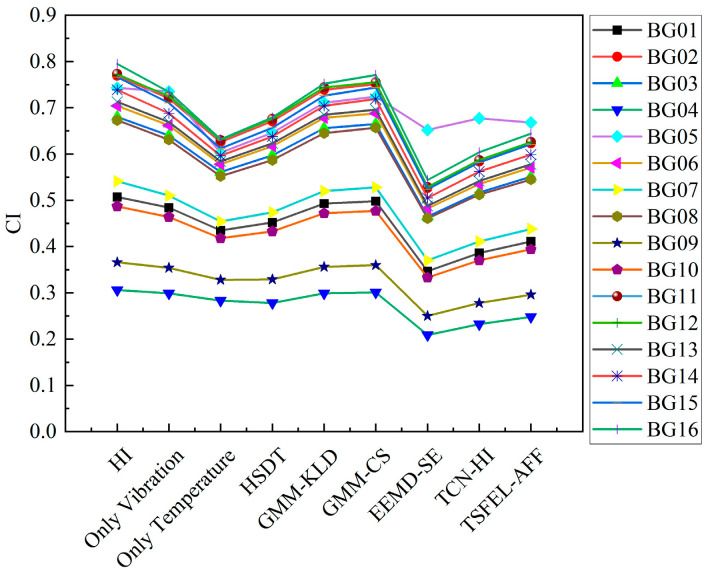
Statistical graph of comprehensive evaluation indicators of HI.

Figure 13 shows the comprehensive index comparison across bearings. The proposed HI achieves the highest average value, confirming enhanced degradation sensitivity and trend characterization capability.

**Figure 14:**
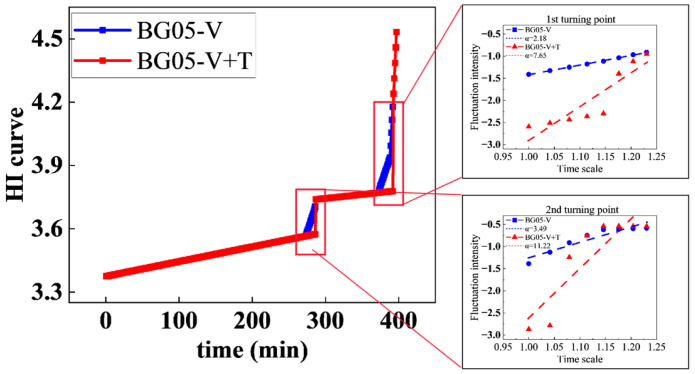
HI curve comparison and DFA analysis.

To further evaluate and compare the health indicator (HI) developed in this study, a comparative analysis was conducted with the HI curve constructed solely from vibration signals, using bearing BG05 as a case study. The results are presented in Figure 14. It can be observed that the vibration–temperature fused HI curve exhibits clearer stage demarcation. Detrended fluctuation analysis (DFA) was applied to compare the scaling exponents α at two critical transition points of health state changes. The analysis reveals that the fused vibration–temperature HI demonstrates higher α values, indicating more pronounced long-term trends in health state evolution and enhanced identifiability of lifespan segmentation. The comprehensive metrics in Figure 13, derived from all 16 test bearings, demonstrate our method’s consistent performance across different bearing units. While BG05 serves as our detailed example, these aggregate results confirm the methodology’s generalizability.

## 5. Conclusions

This study addresses the limitations of single vibration signals in rolling bearing health monitoring by proposing a multi-source signal fusion and adaptive optimization-based approach. By integrating vibration and temperature signals, a multidimensional time–frequency feature set is constructed, and the adaptive feature fusion–auto-associative kernel regression (AFF–AAKR) strategy is employed to significantly enhance the health indicator’s (HI) degradation characterization capability. The proposed method outperforms conventional approaches with a comprehensive index (CI), representing our integrated performance metric combining monotonicity, correlation, and robustness indicators, as introduced in Section 1.

While this study demonstrates the efficacy of vibration–temperature fusion for degradation identification, future work will focus on the following:

(1) Establishing a bearing testbed with seeded faults (e.g., artificial spalls, cracks) to acquire degradation data with physically confirmed transition points;
(2) Validating the method on field data from gas-turbine bearings through industry collaborations, with failure modes documented via post-mortem analysis;
(3) Incorporating bearing cage slip monitoring and adaptive CI weighting to enhance early fault detection under diverse operating conditions;
(4) Developing a degradation-integrated simulation model for generating synthetic vibration data with programmable defect modes, leveraging dynamic modeling principles for non-stationary signals to address limited physical test data scenarios.

## Figures and Tables

**Table 1:** Time-domain features.

Features	Abbreviation	Definition
Mean	Mean	$\frac{1}{N}\sum_{i=1}^{N}x_{i}$
Variance	Var	$\frac{1}{N}\sum_{i=1}^{N}(x_{i}-\frac{1}{N}\sum_{i=1}^{N}x_{i}{)}^{2}$
Standard Deviation	Std	$\sqrt{\frac{1}{N}\sum_{i=1}^{N}(x_{i}-\frac{1}{N}\sum_{i=1}^{N}x_{i}{)}^{2}}$
Peak-to-Peak Value	P2P	$\max(x_{i})-\min(x_{i})$
Root Mean Square Value	RMS	$\sqrt{\frac{1}{N}\sum_{i=1}^{N}{x_{i}}^{2}}$
Absolute Mean	Absmean	$\frac{1}{N}\sum_{i=1}^{N}\left|x_{i}\right|$
Skewness	Skew	$\frac{\frac{1}{N}\sum_{i=1}^{N}(x_{i}-\frac{1}{N}\sum_{i=1}^{N}x_{i}{)}^{3}}{{X_{\mathrm{Std}}}^{4}}$
Kurtosis	Kur	$\frac{\frac{1}{N}\sum_{i=1}^{N}(x_{i}-\frac{1}{N}\sum_{i=1}^{N}x_{i}{)}^{4}}{{X_{\mathrm{Std}}}^{4}}$
Crest Factor	CF	$\frac{\max(\left|x_{i}\right|)}{\sqrt{\frac{1}{N}\sum_{i=1}^{N}x_{i}^{2}}}$
Wave Factor	WF	$\frac{\sqrt{\frac{1}{N}\sum_{i=1}^{N}x_{i}^{2}}}{\frac{1}{N}\sum_{i=1}^{N}\left|x_{i}\right|}$

$x_{i}$ represents the signal sampling points; N denotes the total number of sampling points.

**Table 2:** Frequency-domain features.

Features	Abbreviation	Definition
Mean Frequency	MF	$\frac{\sum_{k=1}^{M}f_{k}\cdot S\left(f_{k}\right)}{\sum_{k=1}^{M}S\left(f_{k}\right)}$
Frequency-domain RMS	FRMS	$\sqrt{\frac{1}{M}\sum_{k=1}^{M}S{\left(f_{k}\right)}^{2}}$

$f_{k}$: frequency points, $S(f_{k})$: amplitude at the $f_{k}$ frequency point, M: total number of frequency points.

**Table 3:** Experimental bearing structural parameters.

Inner Diameter (mm)	Outer Diameter (mm)	Number of Rolling Elements	Rolling Element Diameter (mm)
30	62	14	7.5

**Table 4:** Sensor parameters.

Test Category	Type	Measurement Range	Sampling Rate
Vibration	Acceleration	0~1000 g	25,600 Hz
Temperature	Piezoelectric	Rt-250 °C	1 Hz

**Table 5:** Experimental operating conditions.

Load	Speed	Lubrication Method	Oil Supply Temperature
10,000 N	18,000 rpm	Jet Lubrication	Room Temperature

**Table 6:** Feature clustering results.

Cluster	Included Features	Feature Type
C1	Std, P2P, RMS, Var	High-Sensitivity Time-Domain Statistical Features
C2	E4, E5, E6, E7, E2, E8, E3, E1	Low-Sensitivity Frequency-Domain Features
C3	AbsMean, Mean	Low-Sensitivity Distribution Shape Features

**Table 7:** Comparison of feature weights within clusters.

Cluster	Feature	Initial Weight	Regularized Weight (λ = 0.1)
C1	Std	0.285	0.255
	P2P	0.280	0.250
	RMS	0.225	0.195
	Var	0.210	0.180
C2	E1	0.180	0.160
	E2	0.150	0.130
	E3	0.140	0.120
	E4	0.130	0.110
	E5	0.125	0.105
	E6	0.120	0.100
	E7	0.115	0.095
	E8	0.110	0.090
C3	Absmean	0.620	0.580
	Mean	0.380	0.340

**Table 8:** Results of cluster representation selection.

Cluster	Representative Feature	Regularized Weight	Total Mutual Information	Threshold τ = 1.0 Met?
C1	Std	0.255	0.92	Yes
C2	E1	0.160	0.88	Yes
C3	AbsMean	0.580	0.10	Yes

**Table 9:** Fsec of 16 bearings.

Bearing	Fsec	Bearing	Fsec
BG01	{Std, E1, AbsMean}	BG09	{RMS, Kur, E2}
BG02	{P2P, Means, E4}	BG10	{CF, AbsMean, Skew}
BG03	{E4, Means, Std}	BG11	{CF, Skew, WF}
BG04	{P2P, Skew, MF}	BG12	{Std, Skew, FRMS}
BG05	{Var, Skew, MF}	BG13	{Skew, P2P, WF}
BG06	{Var, Skew, E4}	BG14	{P2P, Skew, WF}
BG07	{CF, Std, P2P}	BG15	{CF, Skew, Kur}
BG08	{Std, P2P, WF}	BG16	{Std, Skew, E4}

**Table 10:** Information entropy and weight table of vibration and temperature.

	Vibration	Temperature
Information Entropy	0.911	0.859
Weight	0.387	0.613

## Data Availability

The data generated or analyzed during this study are available from the corresponding author upon reasonable request.
